# Possible therapeutic effects of *Nigella sativa* and its thymoquinone on COVID-19

**DOI:** 10.1080/13880209.2021.1931353

**Published:** 2021-06-10

**Authors:** Mohammad Reza Khazdair, Shoukouh Ghafari, Mahmood Sadeghi

**Affiliations:** a Pharmaceutical Science and Clinical Physiology, Cardiovascular Diseases Research Center, Birjand University of Medical Sciences, Birjand, Iran; b Infectious Diseases Research Center, Birjand University of Medical Sciences, Birjand, Iran; c Medical Toxicology and Drug Abuse Research Center (MTDRC), Birjand University of Medical Sciences, Birjand, Iran

**Keywords:** Immunomodulation, anti-inflammatory, antiviral effects, medicinal plant

## Abstract

**Context:**

COVID-19 is a novel coronavirus that causes a severe infection in the respiratory system. *Nigella sativa* L. (Ranunculaceae) is an annual flowering plant used traditionally as a natural food supplement and multipurpose medicinal agent.

**Objective:**

The possible beneficial effects of *N. sativa*, and its constituent, thymoquinone (TQ) on COVID-19 were reviewed.

**Methods:**

The key words including, COVID-19, *N. sativa*, thymoquinone, antiviral effects, anti-inflammatory and immunomodulatory effects in different databases such as Web of Science (ISI), PubMed, Scopus, and Google Scholar were searched from 1990 up to February 2021.

**Results:**

The current literature review showed that *N. sativa* and TQ reduced the level of pro-inflammatory mediators including, IL-2, IL-4, IL-6, and IL-12, while enhancing IFN-γ. *Nigella sativa* and TQ increased the serum levels of IgG1 and IgG2a, and improved pulmonary function tests in restrictive respiratory disorders.

**Discussion and conclusions:**

These preliminary data of molecular docking, animal, and clinical studies propose *N. sativa* and TQ might have beneficial effects on the treatment or control of COVID-19 due to antiviral, anti-inflammatory and immunomodulatory properties as well as bronchodilatory effects. The efficacy of *N. sativa* and TQ on infected patients with COVID-19 in randomize clinical trials will be suggested.

## Introduction

*Nigella sativa* L. (Ranunculaceae), or black seed, has been used traditionally as a food additive and spice (Khazdair, Anaeigoudari, Hashemzehi et al. 2019). The use of plants and botanical compounds for immune enhancement has been reported by several recent studies and traditional medicine sources (Roxas and Jurenka 2007). *Nigella sativa* is among the most commonly used herbal plants practiced in Iranian traditional medicine (Gilani et al. 2004). *Nigella sativa* is traditionally used for the treatment of various types of disorders including diabetes, cough, fever, eczema, bronchitis, and influenza (Ali and Blunden 2003). Pharmacological effects of *N. sativa* including the anti-inflammatory, antioxidant (Mohebbatia, Khazdair, Karimia et al. 2017; Bordoni et al. 2019), antimicrobial (Emeka et al. 2015), neuro-protective (Mohebbatia, Khazdairb, Hedayatia et al. 2017; Khazdair, Anaeigoudari, Hashemzehi et al. 2019), and reno-protective properties (Mohebbati et al. 2017) were reported.

COVID-19 is an enveloped virus with a single-stranded RNA genome, and the third known coronavirus after severe acute respiratory syndrome (SARS) and Middle East respiratory syndrome coronavirus (MERS-CoV) (Malik et al. 2020). Infection with COVID-19 leads to severe respiratory disorders and pneumonia-like symptoms in humans (Shanmugaraj et al. 2020). COVID-19 has high transmissibility and infectivity compared with SARS and MERS (Liu et al. 2020). Traditionally, it has been known that some medicinal plants and their products possess immune-regulatory properties. The isolation of plant bioactive components occurred in the nineteenth century (Phillipson 2001; Khazdair, Anaeigoudari, Kianmehr et al. 2019).

It has been reported that about 64% of the world population use herbal remedies for the treatment of various disorders (Farnsworth 2008). Moreover, nearly 50% of synthetic drugs are derived from phytochemicals (Newman and Cragg 2012). Herbs synthesize chemicals as a part of their defence system to combat pathogens; and a considerable number of such compounds are effective anti-infective agents. For example, naturally occurring hydroxylated phenols and flavonoids are effective against infections (Dixon et al. 1983). Alkaloids, as the most common plant-based bioactive metabolites, as well as flavonoids have antifeedant and larvicidal effects (Levin and York 1978).

Natural products and essential oils are well recognized for their antiviral, anti-inflammatory and immuno-modulatory activities (Asif et al. 2020; Kumar et al. 2020). It has been reported that various monoterpenoid phenols obtained from plants including carvacrol have the potential to inhibit the binding of viral spike (S) glycoprotein to the host cell (Kulkarni et al. 2020). Also carvacrol can inhibit ACE2 activity and suggested that it may block the host cell entry of SARS-CoV-2 (Abdelli et al. 2020).

This review tries to explain the traditional and new pharmacological properties of *N. sativa* and its main ingredient, thymoquinone on COVID-19 induced infection in the respiratory system based on anti-inflammatory effects and antiviral activities.

## Methods

Data of the current study were obtained from the most popular scientific databases, Web of Science (ISI), PubMed, Scopus, and Google Scholar by searching keywords: ‘COVID-19’ and ‘*Nigella sativa*’ or ‘thymoquinone’ or ‘Antivirus effects’ in the title and ‘inflammatory lung diseases’ or ‘immunomodulatory effects’ in the title or abstract. Relevant published articles in the English language up to February 2021 were included. All studies evaluating the effects of *N. sativa* or thymoquinone on viral diseases, and inflammatory lung diseases were included. Articles with insufficient information and in another language were excluded from the review.

## Results

### The potential immunomodulatory effects of medicinal herbs

Immunotherapy is characterized as an approach to disease management by producing or enhancing an immune response to a present disorder (Vanderlugt and Miller 2002). Cytokines such as, interleukins (IL), chemokines, interferons (IFN), and tumour necrosis factors (TNF) are small, non-structural proteins, which have multitude effects in various organs (Dinarello 2007). The pro-inflammatory mediators include IL-17, IL-1β, and TNF-α, and anti-inflammatory mediators include, IL-10, and IL-1ra (Su et al. 2012). The pathogenic roles of cytokines including; IL-6, IL-10, IL-17, IL-23, IFN-α and IFN-γ in a heterogenic autoimmune inflammatory disease such as systemic lupus erythematosus (SLE) is shown (Su et al. 2012). The roles of Th2 cytokines such as IL-4 in the pathogenesis of asthma is also reported (Steinke and Borish 2001).

Deregulation of the immune system has been known as the main cause of many diseases; thus, management of immune responses could be a beneficial therapeutic strategy for the treatment of these diseases. Some medicinal plants might affect the functions of the immune system by modulation of the production/release of immune-globulins and cytokines, immune cells activities, and cellular coreceptor expression (Das et al. 2004).

### Immune system response to COVID-19

As antigens, viruses stimulate humoral and cellular immune responses. The induction of the immune system response to a virus is mediated by virus-specific T and B cells (Cox and Brokstad 2020). The pattern of antibody production, especially the production of immunoglobulins M and G (IgM and IgG), against SARS-CoV-2 is similar to common acute viral infections (Li et al. 2003). The number of CD4^+^ and CD8^+^ T cells as humoral responses significantly reduced in the peripheral blood of infected patients with SARS-CoV2 (Xu et al. 2020). Similarly, reduction of CD4^+^ and CD8^+^ T cells in the acute phase of infection with SARS-CoV is also associated with reduction in the number of CD4^+^ and CD8^+^ T cells (Fan et al. 2009). Moreover, CD8^+^ T cells also showed a similar effect in infected mice with MERS-CoV (Zhao et al. 2014).

Cytokine storm is one of the main mechanisms for acute respiratory distress syndrome (ARDS), the systemic inflammatory response resulting from the release of large amounts of pro-inflammatory mediators including IFN-α, IL-1b, IL (6 − 12 − 18 and 33), TNF-α, TGFβ, etc. and chemokines by immune effector cells in SARS-CoV infection (Cameron et al. 2008; Huang et al. 2020). Patients with MERS-CoV infection showed elevated levels of pro-inflammatory mediators in the serum similar to infection with SARS-CoV (Min et al. 2016). The cytokine storm will be started by the immune system to cause ARDS and various organs to failure, which may be lead to death in severe cases infected with SARS-CoV-2 and MERS-CoV (Xu et al. 2020).

### Pharmacological effects of *N. sativa*

*Nigella sativa* is widely grown in the Mediterranean region, the west of Asia, Middle East, southern Europe, and north Africa (Tembhurne et al. 2014). *Nigella sativa* seed has been traditionally used for the treatment of fever, infection, chest congestion, inflammation, cough, bronchitis, asthma, chronic headache, dysmenorrhoea, obesity, diabetes, flatulence, and diarrhoea (Durmuskahya and Ozturk 2013; Nasir et al. 2014). The active ingredient of *N. sativa* seed is mainly thymoquinone (TQ) which showed anti-inflammatory effects by suppression of prostaglandins and leukotrienes as inflammatory mediators (Hajhashemi et al. 2004). Antioxidant and anti-epileptic, as well as anti-Alzheimer’s and anti-Parkinson’s disease effects of *N. sativa* and TQ, were previously reported (Khazdair 2015).

### Antiviral activities of *N. sativa* and TQ

Antiviral effect of *N. sativa* oil in murine cytomegalovirus (MCMV) model was investigated by administration of *N. sativa* oil (100 μg/100 μL, i.p.) to BALB/c mice. Treatment with *N. sativa* oil significantly reduced the load of virus in spleen and liver 3 days after infection compared to the control group. The antiviral effect of the plant oil accorded with increasing serum level of IFN-γ and increasing numbers of CD4^+^ helper T cells. Moreover, the titre of virus was undetectable in liver and spleen after 10 days while it was detectable in control mice (Salem and Hossain 2000). The antiviral effect of plant oil is related to the increasing response of CD4 cells. The effects of *N. sativa* oil on pathogenesis and immune response of H9N2 avian influenza virus (H9N2 AIV) in infected turkeys showed positive results. Turkeys fed with diets containing 2%, 4% and 6% of *N. sativa* oil significantly decreased mortality rate along with an increased in the body weight of turkeys. Turkeys fed with *N. sativa* (6%) had significantly lower titre of virus compared to the control group. Moreover, *N. sativa* increased the mRNA expression of IFN-γ compared to the control group. The enhancement of antibody titre against H9N2 AIV in turkeys fed with *N. sativa* showed the immune regulatory effects of the plant. Additionally, treatment of (H9N2 AIV) infected turkeys with TQ and curcumin significantly increased expression of IFN-γ and antibody titre against H9N2 AIV in birds. TQ also reduced virus shedding and enhanced immune responses in treated animals that lead to suppress pathogenesis of H9N2 viruses (Umar et al. 2016).

Treatment of a hepatitis C virus (HCV) infected patient, who was not eligible for IFN-α therapy, with capsules of *N. sativa* oil (450 mg) for 3 months (three times daily), significantly decreased the viral load and also improved oxidative stress due to augmented total antioxidant activity. Moreover, *N. sativa* oil improved red blood cells (RBC), platelet counts, total protein, and albumin in HCV-infected patient (Barakat et al. 2013).

The beneficial effects of *N. sativa* oil on immunity in microbial infection could be augmented by Zinc (Zn) supplement. The possible therapeutic effects of *N. sativa* and Zn supplements to treat COVID-19 was suggested (Rahman 2020).

The potential efficacy of *N. sativa* oil (500 mg soft-gel capsules) one capsule orally twice daily for 10 days plus standard of care treatment on the outcomes of patients with mild COVID-19 was investigated (Koshak et al. 2020).

The pharmacological properties of *N. sativa* seed and TQ including immunomodulatory, antioxidant and anti-inflammatory active and their potential therapeutic strategy against COVID‐19 were reviewed (Islam et al. 2020). It has been reported that some natural products such as TQ have high to a moderate binding affinity to the heat Shock Protein A5 (HSPA5) substrate-binding domain β (SBDβ), which reported to be the recognition site for the SARS-CoV-2 spike. This natural compound may be used to reduce the risk of COVID-19 or possibly to treat the disease, especially in high-risk people (Elfiky 2020).

It has been reported that the binding affinity of a *N. sativa* constituent, dithymoquinone (DTQ), was higher than a positive control (chloroquine), which has the high potential affinity binding at SARS–CoV-2:ACE_2_ interface. Then, it could be predicted as an inhibitor to disrupt viral-host interactions (Ahmad et al. 2020).

The constituents of *N. sativa* such as, α-hederin, thymohydroquinone, and TQ have efficiently binding to ACE_2_ and potential therapeutic effects of these bioactive components to combat COVID-19 in-silico study was suggested (Jakhmola Mani et al. 2020). The affinity of TQ on SARS–Cov-2 E protein and inhibitory effects on E protein ion channel were showed in the molecular docking study (Mohideen 2021). It has been reported that TQ may inhibit SARS–CoV‐2 and interfere with its binding to ACE2 receptors in molecular docking studies (Bouchentouf and Missoum 2020; Sekiou et al. 2020). This can prevent virus entry and replication inside the host cell. The results of these studies demonstrate the potential of TQ on virus machinery as well as virus entry and the replication in the host cells. Antiviral effects of *N. sativa* and TQ are shown in Table 1.

**Table 1.** Antiviral effects of *N. sativa* and TQ.

Type of plant Extract	Effective doses	Model of study	Effects	Reference
*N. Sativa* oil	100 μg/100 μL	MCMV	Inhibited the virus titres in spleen and liver 3 and significantly reduced the viral load in the liver and spleen. Raising IFN-γ serum level and increasing numbers of CD4+ helper T cells	Salem and Hossain (2000)
Fed diets of *N. Sativa*	2%, 4% and 6%	H9N2 AIV	Significantly increased the body weight and reduced mortality was observed in turkeys. Significantly lower virus titre than those in control group. Moreover, fed diets of *N. sativa* increased the expression levels of IFN-γ mRNA compared to the control group.	Umar et al. (2016)
*N. sativa* oil	Capsule (450 mg)	HCV patients	Significant decreased the viral load and also improvement of the oxidative stress due to augmented total antioxidant activity. N. sativa oil also improved RBC, platelet counts, total protein and albumin in HCV patients	Barakat et al. (2013)
Fed diets of TQ	5 g/kg	H9N2 AIV	Significantly increased antibody titre against H9N2 and increased gene expression of IFN-γ. TQ also reduced virus shedding and enhanced immune responses in treated animals that lead to suppress pathogenesis of H9N2 viruses	Umar et al. (2016)

MCMV: murine cytomegalovirus; H9N2 AIV: H9N2 avian influenza virus; HCV: hepatitis C virus.

## *Immunomodulatory and anti-inflammatory effects of* N. sativa *and TQ*

### Nigella sativa

This herb has been used as a safe herbal food against inflammatory diseases including asthma, allergy, and metabolic syndrome due to its immunomodulatory effects and low cytotoxicity (Gholamnezhad et al. 2015). Ethanol extract of *N. sativa* seed (1000 µg/mL) reduced the secretion of IL-4 in phytohemagglutinin (PHA) and concanavalin A (Con A)-stimulated splenocytes. In addition, *N. sativa* seed extracts (500 and 1000 µg/mL) decreased IFN-γ secretion in stimulated and non-stimulated splenocytes (Gholamnezhad et al. 2015). The effects of *N. sativa* seed aqueous extract (1, 10, 50, and 100 µg/mL) on splenocytes proliferation, the function of macrophages in BALB/c mice, and natural killer (NK) activity of C57/BL6 showed significant and dose-dependently elevation of the splenocytes proliferation (Majdalawieh et al. 2010). The pattern of cytokine secretion by splenocytes was changed by the plant extract in favour of Th_2_ pathway. The aqueous extract of *N. sativa* seed (50 and 100 µg/mL) significantly enhanced the secretion of IL-4 and IL-10 but suppressed TNF-α, IL-6, and NO by primary macrophages. In addition, the plant extract increased NK cells cytotoxic activity against YAC-1 tumour cells which suggested an antitumor activity for the plant (Majdalawieh et al. 2010).

Nevertheless, cytokines production by splenic mononuclear cells (MNCs) of the treated ovalbumin (OVA)-sensitized mice with *N. sativa* oil (252 mg, p.o.) were not significantly different from that of the non-treated group. These results indicated that *N. sativa* oil has no immune regulatory effect on Th_1_ and Th_2_ cell responsiveness to allergen stimulation (Büyüköztürk et al. 2005).

*Nigella sativa* oil exhibits airway anti-inflammatory and immune-regulatory effects which may support its use for treatment of allergic asthma. The eosinophil count in peripheral blood, IgG1 and IgG2a levels, cytokines profile including, IL-2, IL-10, IL-12, and IFN-γ levels, and also inflammatory cells in the lung tissue were significantly decreased by the plant oil in a mouse model of allergic asthma. The plant showed comparable immunomodulatory properties with those of dexamethasone except for its effect on IFN-γ level (Abbas et al. 2004).

*Nigella sativa* seed ethanolic extract supplementation (200 mg/kg/day, p.o.) in control, moderately trained, and over-trained rats changed the cytokines profile. Immediately after exercise, IL-6, IL-10, and TNF-α were increased while IL-4 was decreased in rats’ serum. Moreover, IFNγ/IL-4 ratio significantly increased in animals treated with the plant extract (Gholamnezhad et al. 2014).

Pre-treatment of OVA-sensitized guinea pigs with *N. sativa* seed hydro-ethanolic extract (1.25 and 2.50 g/L, p.o.) reduced IL-4 level, but increased the level of IFN-γ and ameliorated almost all lung histological changes in the sensitized animals (Boskabady, Keyhanmanesh et al. 2011). In another experiment, *N. sativa* hydro-ethanolic extract (0.08 g, p.o.) decreased neutrophil numbers and restored IL-4 and IFN-γ levels in sulphur mustard (40 mg/m^3^) exposed guinea pigs (Boskabady, Keyhanmanesh et al. 2011; Boskabady, Vahedi et al. 2011).

Administration of *N. sativa* seeds volatile oil (2.5 µL ≅ 2.10 µg, intramuscular (i.m.), twice a week for 30 days) in rats significantly reduced antibody titre (1280 versus 2560) compared to the control animals. Furthermore, the splenocytes and neutrophils counts were significantly decreased, but peripheral lymphocytes and monocytes were increased in the experimental animals that received *N. sativa* seeds volatile oil (Islam et al. 2004). Serum protein and total immunoglobulin levels of fish fed with diets containing 1, 2.5 and 5% of *N. sativa* oil for 21 days were significantly increased. Furthermore, haematocrit level was significantly increased in group fed with *N. sativa* (5%) compared to the control group (Dorucu et al. 2009).

IL-1β and IL-4 levels were increased following addition of *N. sativa* seed aqueous extract (1 and 2 µg/mL) to culture medium of non-activated peripheral blood mononuclear cells (PBMC) and allogeneic cells. Whole *N. sativa* proteins (0.1, 1 or 10 µg/mL) suppressed the production of IL-8 in non-stimulated as well as pokeweed mitogen-activated PBMC cells. Whole soluble *N. sativa* seed extract (2 µg/mL) also increased TNF-α production. Furthermore, fractionated extract of *N. sativa* was less effective than whole *N. sativa* proteins (Haq et al. 1999).

Aqueous *N. sativa* seed extract (50 µg/mL) suppressed lymphocytes response to all mitogens and allogeneic cells. Also, *N. sativa* extract (0.5 µg/mL) stimulated lymphocytes response to allogeneic cells. Moreover, below-10-kDa fraction of *N. sativa* stimulated the production of IL-1β and IL-3 by human lymphocytes without need for any mitogen or other human allogeneic cells. The most marked increase in IL-3 production was noted when *N. sativa* extract (0.5 µg/mL) was added to lymphocytes culture. However, *N. sativa* extract did not effect on IL-2 secretion by mitogen-activated lymphocytes (Haq et al. 1995). In autoimmune encephalomyelitis (AE)-induced in Wistar rats, whole *N. sativa* seed (2.8 g/kg, bw) reduced the expression of transforming growth factor beta 1 (TGF β1) and increased remyelination in the cerebellum (Noor et al. 2015). Oral administration of hydro-ethanolic extract of *N. sativa* seed (100, 200, 400 mg/kg, i.p.) on LPS (1 mg/kg, i.p.)-induced lung injury in rats, decreased the total and different WBC counts as well as oxidative stress biomarkers in the bronchoalveolar lavage fluid (BALF) and serum. Furthermore, treatment with *N. sativa* extract dose-dependently reduced TGF-β1, IFN-γ, PGE2, IL-4 levels in the BALF as well as pathological changes in the lung (Mokhtari-Zaer et al. 2020).

In a clinical study, dietary supplementation with *N. sativa* oil improved the immune response in healthy elderly subjects (Salem 2005). Prostaglandin E_2_ production was significantly reduced in individuals who received *N. sativa* oil (750 mg) compared to the placebo treated group (750 mg soybean oil) (Wu et al. 1999).

Administration of *N. sativa* oil capsules (40–80 mg/kg/day, p.o.) in patients with allergic rhinitis, atopic eczema and asthma significantly reduced the levels of IgE, endogenous cortisol and eosinophil count in plasma and urine compared to their pre-treatment values (Kalus et al. 2003).

Together, the results of different studies indicated that *N. sativa* influences serum immunoglobulins, antibody titre, eosinophil count, cytokine profiles, and Th_1_/Th_2_ balance. Therefore, *N. sativa* could be applied for the treatment of inflammatory diseases such as allergy and asthma. Immunomodulatory effects of *N. sativa* are summarized in Table 2.

**Table 2.** Anti-inflammatory and immuno-modulatory effects of *N. sativa*.

Type of plant extract	Effective doses	Model of study	Effects	Reference
Ethanolic extract	1000 µg/mL	Splenocytes cells	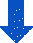 IL-4 and IFN-γ	Gholamnezhad et al. (2015)
Aqueous extract	2 µg/mL	PBMC cells	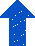 TNF-α, IL-1β and IL-4 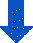 IL-8	Haq et al. (1999)
	100 µg/mL	Splenocytes cells	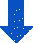 IL-6, TNF-α, and NO 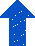 IL-4 and IL-10	Majdalawieh et al. (2010)
	0.5 µg/mL	Human lymphocyte	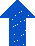 IL-1β and IL-3	Haq et al. (1995)
Ethanolic extract	200 mg/kg, p.o.	Rat	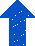 IL-6, IL-10 and TNFα 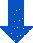 IL-4	Gholamnezhad et al. (2014)
Hydroethanolic	2500 mg/L, p.o.	Guinea pigs	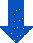 IL-4, 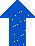 IFN-γ	Boskabady, Keyhanmanesh et al. (2011)
	80 mg/L, p.o.	Guinea pigs	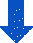 Neutrophil number and restored the IL-4 and IFN-γ changes	Boskabady, Vahedi et al. (2011)
Volatile oil	2.10 µg, i.m	Rat	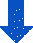 Splenocytes and neutrophils counts 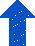 Peripheral lymphocytes and monocytes	Islam et al. (2004)
Aqueous extract	2.8 g/kg,bw	Rat	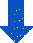 The expression of transforming growth factor beta 1 (TGF β1)	Noor et al. (2015)
Diet of *N. sativa*	5%	Rainbow trout	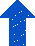 Serum protein and total Ig levels	Dorucu et al. (2009)
Supplement of *N. sativa* oil	10 mg/kg, p.o.	Human	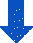 Production of prostaglandin E_2_	Salem (2005)
Capsule of *N. sativa* oil	40 mg/kg/day, p.o.	Human	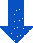 IgE, eosinophil count, endogenous cortisol in plasma and urine	Kalus et al. (2003)

IL: interleukins; IFN-γ: interferon gamma; TNF-α: tumour necrosis factors alpha; PBMC: peripheral blood mononuclear cell; TGF β1: transforming growth factor beta 1; Ig: immunoglobulin.

### Thymoquinone (TQ)

Treatment of LPS-activated mast cells with TQ (10 μM) restored LPS-induced changes in IL-5 and IL-13 at mRNA and protein levels but did not affect IL-10 production. In addition, TQ inhibited globin transcription factor (GATA) binding at the IL-5 promoter induced by LPS stimulation (El Gazzar 2007). In addition, TQ treatment (1–20 μM) significantly inhibited the release of IL-10, IL-12, and TNF-α from LPS-induced dendritic cells (DCs) and suppressed phosphorylation of pro-survival factors protein kinase B (AKT) and extracellular signal-regulated protein kinases 1 and 2 (ERK1/2), but induced caspase-3 and caspase-8 activity in DCs (Xuan et al. 2010). TQ (0–25 μM) also dose-dependently inhibited TNFα-induced nuclear factor-κB (NF-κB) activation via inhibition of NF-κB kinase (IKK) activity, and subsequently suppressed phosphorylation and activation of NF-κBα (IκBα) (Sethi et al. 2008).

The gene expression of NF-κB regulated of anti-apoptotic factors such as inhibitor of apoptosis proteins (IAP)-1, IAP2, XIAP Bcl-2, and Bcl-xL, and angiogenic (MMP-9 and VEGF) gene products were also down regulated in human myeloid leukaemia cell line (KBM-5) by TQ (Sethi et al. 2008). The gene expression of IL-1β, TNF-α, monocyte chemoattractant protein-1 (MCP-1), and cyclooxygenase-2 (COX-2) in pancreatic ductal adenocarcinoma (PDA) cells were significantly reduced by TQ (25–75 μM). TQ also showed an incisory effect on TNF-α mediated of NF-κB activation in placenta-derived cells (PDA) but decreased the transport of NF-κB from the cytosol to the nucleus (Chehl et al. 2009).

The prophylactic effect of TQ (3 mg/kg, i.p.) on the levels of IL-4 and IFN-γ was studied in sensitized guinea pigs and the results showed decreased IL-4 but increased IFN-γ levels (Rana Keyhanmanesh et al. 2014).

Administration of TQ (3 mg/kg, i.p.) decreased the production of leukotriene B4 (LTB4) and leukotriene C4 (LTC4) in the BALF of mice. Furthermore, the levels of IL-4, IL-5, and IL-13 were also significantly decreased while IL-10 was increased when TQ administered before OVA challenge (El Gazzar, El Mezayen, Nicolls et al. 2006). Similarly, TQ (3 mg/kg, i.p.) significantly decreased elevated serum levels of IgE and IgG1 and also inhibited allergen induced lung inflammation and production of mucus by goblet cells. TQ also significantly inhibited IL-4, IL-5, and IL-13 but increased IFN-γ production in the BALF. In addition, a small effect of TQ was observed on the production of IL-4 in OVA-stimulated cultured lung cells. These results indicated the effect of TQ on reduction of airway inflammation (inhibition of eosinophil infiltration into the airways) and Th_2_ cytokines productions (El Gazzar, El Mezayen, Marecki et al. 2006). Intraperitoneal administration of TQ (5 or 10 mg/kg) 30 min before LPS injection (1 mg/kg i.p.) decreased the levels of IL-6 and TNF-α in treated rats (Bargi et al. 2017). Orally administration of TQ (10, 20, and 40 mg/kg/day, p.o.) for 14 days after Alzheimer’s disease (AD) induction in rats, decreased amyloid-β (Aβ) formation and accumulation, and also reduced the levels of TNF-α and IL-1β. Furthermore, it significantly down regulated the expression of NF-κB and interferon regulatory factor 3 (IRF-3) mRNAs (Abulfadl et al. 2018).

In diabetic rat mothers, TQ supplementation (20 mg/kg, p.o.) during pregnancy and lactation periods restored IL-2 levels and T cells proliferation and saved both circulating and thymus-homing T cells in the rat offspring (Badr et al. 2011). These results indicated an inhibitory effect for TQ on eosinophilia, Th_2_ cytokines, and allergen-specific antibodies which resulted in reduction of allergen-induced inflammation. These results showed immune-modulatory effect of TQ and suggests its therapeutic value in allergic and immune-deficiency induced disorders.

**Figure 1.**
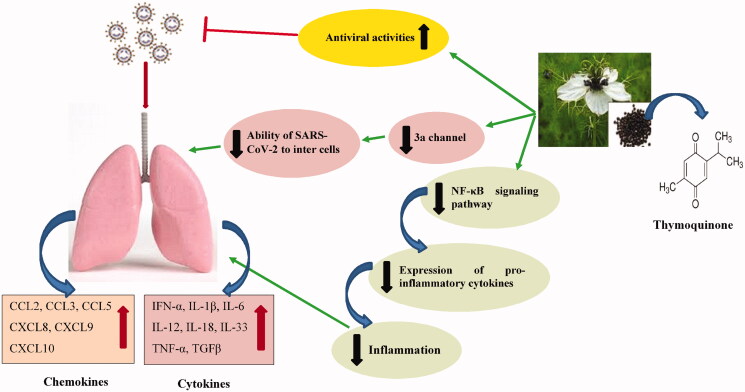
The possible anti-inflammatory and immunomodulatory effects of *N. sativa* and TQ on COVID-19 induced acute respiratory distress syndrome (ARDS).

The relaxation of TQ on trachea smooth muscles due to blockade muscarinic and/or β2 agonistic activity on tracheal tissue from guinea pigs has been reported (Bashir et al. 2020). Anti-inflammatory and immunomodulatory effects of *N. sativa* and TQ are shows in Table 3 and Figure 1.

**Table 3.** Anti-inflammatory and immuno-modulatory effects of TQ.

Bioactive compound	Effective doses	Model of study	Effects	Reference
TQ	10 μM	Mast cells	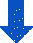 IL-5 and IL-13 mRNA expression	El Gazzar (2007)
	20 μM	Dendritic cells	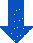 IL-10, IL-12, and TNFα 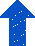 Caspase 3 and caspase 8	Xuan et al. (2010)
	25 μM	KBM-5 cells	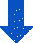 NF-κB activation, anti-apoptotic, and angiogenic gen	Sethi et al. (2008)
	75 μM	(PDA) cells	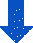 IL-1β, TNFα, MCP-1, and COX-2	Chehl et al. (2009)
	3 mg/kg, i.p.	Guinea pigs	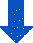 IL-4 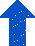 IFN-γ	Rana Keyhanmanesh et al. (2014)
	3 mg/kg, i.p.	Mice	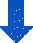 LTB4 and LTC4, IL-4, IL-5 and IL-13 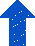 IL-10	El Gazzar, El Mezayen, Marecki et al. (2006)
	3 mg/kg, i.p.	Mice	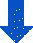 IgE and IgG1, IL-4, IL-5, and IL-13 and IFN-γ	El Gazzar, El Mezayen, Nicolls (2006)
	20 mg/kg, p.o.	Rat	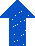 IL-2 and T cell proliferation	Badr et al. (2011)
	5 or 10 mg/kg, i.p.	Rat	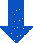 IL-6, TNF-α, and NO metabolites	Bargi et al. (2017)
	10, 20, and 40 mg/kg/day, p.o.	Rat	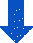 Amyloid-β (Aβ) formation and accumulation, and also decreased TNF-α and IL-1β	Abulfadl et al. (2018)

KBM-5: myeloid leukaemia cell line; PDA: pancreatic ductal adenocarcinoma cells; NF-κB: nuclear factor-κB; MCP-1: monocyte chemoattractant protein-1; COX-2: cyclooxygenase-2; LTB4: leukotriene B4; LTC4: leukotriene C4; NO: nitric oxide.

### Effects of *N. sativa* and TQ on respiratory disorders, the clinical evidences

The prophylactic effect of *N. sativa* boiled extract was shown in asthmatic patients. Administration hydro-ethanolic extract of *N. sativa* seed extract (15 mg/kg/day) for a 3-month period improved respiratory symptoms such as chest wheeze, and pulmonary function test (PFT) values in asthmatic patients compared to the placebo treated group. Furthermore, the need for bronchodilator drugs were decreased in *N. sativa* compared to the placebo-treated patients (Boskabady et al. 2007). The bronchodilatory effect of *N. sativa* seed hydro-ethanolic extract (50 and 100 mg/kg/day, p.o.) in asthmatic patients in comparison with theophylline (6 mg/kg/day) increased pulmonary function tests (PFTs) values, and specific airway conductance (sGaw) compared to the baseline measurements. However, this effect on the most PFT values was less than that of theophylline (Boskabady et al. 2010). Similarly, a 2-month adjuvant treatment with *N. sativa* seed boiled extract (187 mg/kg/day, p.o.) in sulphur mustard poisoned patients showed a decline in the use of bronchodilator drugs compared to the baseline. In addition, the respiratory symptoms and PFT values significantly improved with no adverse effects during the study (Boskabady and Farhadi 2008).

The effect of probiotics or combination with *N. sativa* seed extract (15 mg/kg/day) significantly improved the asthma control test (ACT) score in the patients compared to before intervention (Kardani et al. 2013).

## Conclusions

This review article descriptively highlights the possible effects of *N. sativa* and its major constituent with their underlying mechanism(s) of action on COVID-19. According to our literature survey, *N. sativa* and TQ have various important properties including, antiviral properties, stimulation of humoral and cellular immune responses, modulation of immune responses, improvement of eosinophil counts and IgE serum levels, reduction of pro-inflammatory cytokine (IL-4, IL-1β, IL-6, TGF-β, and IL-17), and enhancement of anti-inflammatory cytokines, IFN-γ and FOXP3. In addition, *N. sativa* and TQ showed relaxant effects on tracheal smooth muscle (*in vitro*) and also improved PFT values in obstructive lung diseases such as asthma. Since, ARDS along with cytokine storm of pro-inflammatory cytokines is the main cause of death among COVID-19 patients and with reference to the anti-inflammatory and immunomodulatory effects of *N. sativa* and TQ, as well as the protective effects on obstructive lung diseases, this herb may be useful for the treatment of COVID-19. However, clinical studies are required to support drug effectiveness.
